# Relationship between thrombomodulin gene polymorphism and susceptibility to venous thromboembolism: A protocol for systematic review and meta-analysis

**DOI:** 10.1097/MD.0000000000025001

**Published:** 2021-03-19

**Authors:** Meihao Wei, Xiaohui Xue, Yipeng Pan, Yan Wu

**Affiliations:** a Department of Nursing; b Department of Gastroenterology; c Department of Pharmacy, Xiashayuan District of Sir Run Run Shaw Hospital affiliated to Medical College of Zhejiang University, Hangzhou, Zhejiang Province, China.

**Keywords:** meta-analysis, polymorphism, protocol, thrombomodulin, venous thromboembolism

## Abstract

**Background:**

Previous studies displayed that thrombomodulin gene polymorphisms are closely associated with venous thromboembolism (VTE), while the results are inconsistent. Therefore, we conducted a meta-analysis to accurately determine the association between thrombomodulin gene polymorphism and the risk of VTE.

**Methods:**

Wanfang, Chinese Biomedical Literature Database, Chinese National Knowledge Infrastructure, the Chongqing VIP Chinese Science and Technology Periodical Database, PubMed, EmBase, and Web of Science databases were searched, and the time to build the database was set until January 2021. The association between thrombomodulin gene polymorphism and the risk of VTE was evaluated. Meta-analysis was performed with STATA 16.0 software, and the odds ratio and its 95% confidence interval were applied to estimate the relationship between thrombomodulin gene polym‘orphism and the risk of VTE.

**Results:**

The results of this meta-analysis will be submitted to a peer-reviewed journal for publication.

**Conclusion:**

This meta-analysis will summarize the relationship between thrombomodulin genepolymorphism and VTE risk.

**Ethics and dissemination:**

Ethical approval was not required for this study. The systematic review will be published in a peer-reviewed journal, presented at conferences, and shared on social media platforms. This review would be disseminated in a peer-reviewed journal or conference presentations.

**OSF REGISTRATION NUMBER:**

DOI 10.17605/OSF.IO/UEHJP.

## 1 Introduction

Venous thromboembolism is caused by genetic, environmental, behavioral, and other factors. Some studies have revealed that about 50% to 60% of the disease is attributed to genetic factors,^[1–3]^ and venous thromboembolism (VTE) is a common fatal disease. The incidence of VTE in China is increasing year by year.^[4]^ However, despite extensive studies, the exact pathogenesis of VTE is still unclear. Inactivity, active cancer, neurological disorders with motor disorders of the lower extremities, trauma/fracture, pregnancy, and oral intake of contraceptive are potential risk factors of VTE.^[5,6]^ However, most individuals with these risk factors do not eventually develop into VTE,^[7]^ which indicates that genetic susceptibility plays an important role in its occurrence and development.

Thrombomodulin is a transmembrane glycoprotein and distributed on the surface of vein, artery, and capillary endothelial cells.^[8–10]^ Thrombomodulin is mainly synthesized by vascular endothelial cells and attached to the surface of endothelial cells, so it plays a very important role in inhibiting blood coagulation and activating fibrinolysis.^[11,12]^ Animal studies have proved that thrombomodulin deficiency or dysfunction and the reduced production of activated Protein C in circulation result in hypercoagulability and prethrombotic state.^[13,14]^ Therefore, perhaps, thrombomodulin plays an important role in the pathogenesis of venous thromboembolism.

Based on the key anticoagulant effects of thrombomodulin, it has been identified that genetic defects in many thrombomodulin genes are associated with the risk of primary and recurrent thrombosis.^[15–17]^ Abnormal expression of thrombomodulin is significant in the occurrence and development of VTE, which clearly indicates that thrombomodulin gene polymorphism can be used as a biomarker to evaluate the risk of VTE. Many studies have explored the relationship between thrombomodulin gene polymorphism and VTE risk.^[15,17–22]^ However, the results of these studies are not consistent. Therefore, we performed a meta-analysis to examine the accurate correlation between thrombomodulin gene polymorphism and VTE risk susceptibility.

## 2 Methods

### 2.1 Study registration

The protocol of this review was registered in OSF (OSF registration number: DOI 10.17605/OSF.IO/UEHJP). It was reported to follow the statement guidelines of preferred reporting items for systematic reviews and meta-analyses protocol.^[23]^

### 2.2 Inclusion criteria

Studies would be included in this meta-analysis on the basis of the following criteria:

1. Types of studies: All case control studies that are associated with the susceptibility of thrombomodulin gene polymorphisms to VTE would be incorporated in our review.
2. Types of participants: Participants suffering from VTE will be included in the meta-analysis. Control subjects should be defined as without VTE or healthy individuals. No restrictions would focus on age, sex, or country.
3. Data of the thrombomodulin gene polymorphism could be available on genotype distributions for the estimation of the odds ratio (OR) with its 95% confidence interval (CI), or adequate data are provided to estimate the corresponding estimate effects (OR, 95% CI).
4. Outcome: VTE risk comparison.

### 2.3 Exclusion criteria

Studies would be excluded from the meta-analysis based on the following criteria: Animal experiments. Controls did not meet Hardy-Weinberg's law of genetic equilibrium. Repeated reporting of the same set of research data. The study was a review, systematic review, meta-analysis, or case series.

### 2.4 Search strategy

Electronic searching would focus on the databases of Wanfang, Chinese Biomedical Literature Database, Chinese National Knowledge Infrastructure, the Chongqing VIP Chinese Science and Technology Periodical Database, PubMed, Web of Science, and Embase, from the inception of database to January 2021. The search strategy in PubMed is illustrated in Table 1, and the corresponding keywords would be applied in other databases. Additionally, references of all retrieved publications were further screened to identify potentially relevant articles.

**Table 1:** Search strategy in PubMed database.

Number	Search terms
#1	Venous Thromboembolism[MeSH]
#2	Thromboembolism, Venous[Title/Abstract]
#3	Venous Thrombosis[MeSH]
#4	VTE[Title/Abstract]
#5	DVT[Title/Abstract]
#6	Deep Vein Thrombosis[Title/Abstract]
#7	Phlebothrombosis[Title/Abstract]
#8	Thrombosis, Deep Vein[Title/Abstract]
#9	Thrombosis, Venous[Title/Abstract]
#10	Deep Venous Thrombosis[Title/Abstract]
#11	Deep-Vein Thrombosis[Title/Abstract]
#12	Deep-Venous Thrombosis[Title/Abstract]
#13	Deep Vein Thromboses[Title/Abstract]
#14	Deep Venous Thromboses[Title/Abstract]
#15	Deep-Vein Thromboses[Title/Abstract]
#16	Deep-Venous Thromboses[Title/Abstract]
#17	Phlebothromboses[Title/Abstract]
#18	Thromboses, Deep Vein[Title/Abstract]
#19	Thromboses, Deep Venous[Title/Abstract]
#20	Thromboses, Deep-Vein[Title/Abstract]
#21	Thromboses, Deep-Venous[Title/Abstract]
#22	Thromboses, Venous[Title/Abstract]
#23	Thrombosis, Deep Venous[Title/Abstract]
#24	Thrombosis, Deep-Vein[Title/Abstract]
#25	Thrombosis, Deep-Venous[Title/Abstract]
#26	Vein Thromboses, Deep[Title/Abstract]
#27	Vein Thrombosis, Deep[Title/Abstract]
#28	Venous Thromboses[Title/Abstract]
#29	Venous Thromboses, Deep[Title/Abstract]
#30	Venous Thrombosis, Deep[Title/Abstract]
#31	Pulmonary Embolism[MeSH]
#32	Pulmonary Thromboembolism[Title/Abstract]
#33	Thromboembolism, Pulmonary[Title/Abstract]
#34	Embolism, Pulmonary[Title/Abstract]
#35	Embolisms, Pulmonary[Title/Abstract]
#36	Pulmonary Embolisms[Title/Abstract]
#37	Pulmonary Thromboembolisms[Title/Abstract]
#38	Thromboembolisms, Pulmonary[Title/Abstract]
#39	PE[Title/Abstract]
#40	or/1-39
#41	polymorph∗[Title/Abstract]
#42	susceptibility[Title/Abstract]
#43	or/41-42
#44	Thrombomodulin[Title/Abstract]
#45	#40 and #43 and #44

### 2.5 Data collection and analysis

#### 2.5.1 Selection of studies

**Figure 1:**
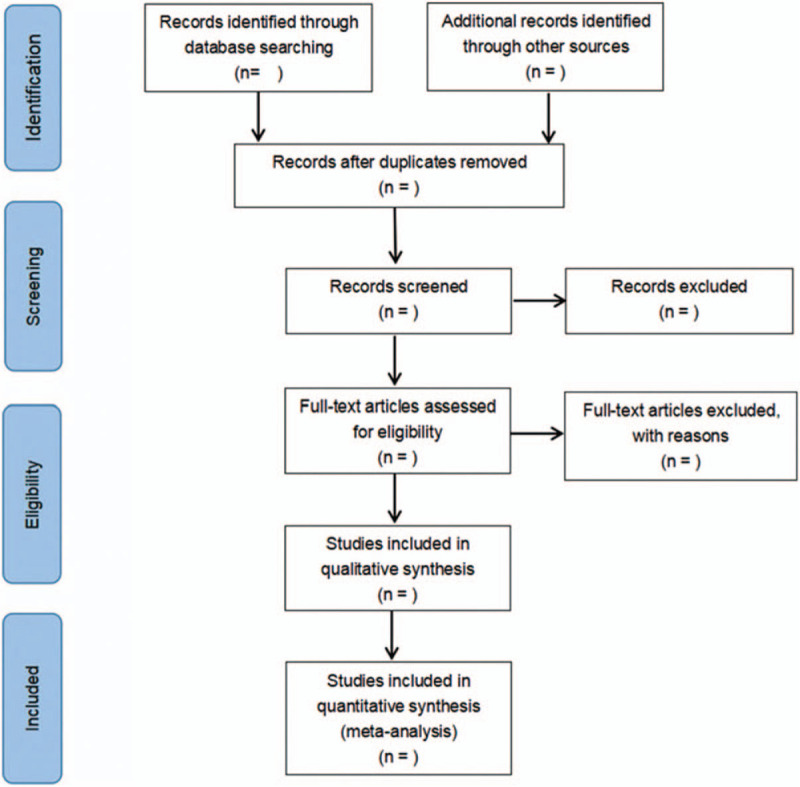
Flow diagram of study selection process.

Data extraction and quality evaluation were performed independently by 2 researchers. In case of missing data in the included studies, the corresponding author was emailed to obtain additional information or raw data. Any disagreements between the 2 researchers were resolved through discussion. The researchers record the reasons to exclude each study in light of the preferred reporting items for systematic reviews and meta-analysis guidelines and report the screening results. The flowchart is demonstrated in Figure 1.

#### 2.5.2 Data extraction

Data extracted from all included studies are as follows: name of the first author; year of publication; country and ethnicity of the participants; number of cases and controls; and genotype distribution of thrombomodulin gene polymorphism in cases and controls. The probability value (*P* value) of Hardy-Weinberg's equilibrium (HWE) was calculated based on the genotype frequency of thrombomodulin polymorphism in the control group. Group discussions are conducted to resolve any disagreements in the extraction process. If the data for a paper is incomplete or unconvincing, we would try to contact the author via email.

#### 2.5.3 Study quality assessment

The quality of all the included studies is evaluated by 2 reviewers independently based on the Newcastle-Ottawa scale (NOS) that is adopted to evaluate the quality of observational studies.^[24]^ The NOS values arrange from 0 to 9. Studies with a score of 6 are considered to be of high quality.^[25]^

#### 2.5.4 Dealing with missing data

The reason for the loss of data in the period of data screening and extraction is identified here. We would attempt to contact the authors if the data of potential studies are insufficient, missing, or vague. These studies would be excluded only if the data are not available through the method described above.

#### 2.5.5 Statistical analysis

Statistical analyses were conducted by using Stata 16.0 (Stata Corporation, College Station, TX). The HWE for control subjects of each study was evaluated by carrying out *χ*^2^ test, and *P* < .05 was regarded as significant disequilibrium. The strength of association between thrombomodulin gene polymorphisms and the susceptibility of cancer was assessed by computing the crude ORs with 95% CIs. The pooled ORs were conducted for 4 genetic models (allelic genetic model: T versus C; recessive genetic model: TT versus CT + CC; dominant genetic model: TT + CT versus CC; and additive model: TT versus CC. T and C represent the mutant allele and the wild-type allele, respectively). The significance of the pooled ORs is determined by *Z* test, and *P* < .05 is considered statistically significant. *χ*^2^ test-based Q statistic and I^2^ would be applied to assess the overall heterogeneities. If I^2^ value < 50% and *P* > .1, heterogeneity is deemed to be low, and a fixed-effect model would be selected for data integration. Otherwise, a random-effect model could be adopted.

#### 2.5.6 Assessment of heterogeneity

Heterogeneity among the included studies will be evaluated by I^2^ statistic. A fixed-effects or random-effects model is utilized to measure pooled OR in the absence or presence of heterogeneity, respectively. When a set of studies exhibit an obvious heterogeneity, factors, including the characteristics of patients and the variation degree in exposure leading to the heterogeneity, should be discussed. Subgroup analysis and sensitivity analysis would be conducted to explore potential sources of heterogeneity across studies when statistical heterogeneity is detected.

#### 2.5.7 Subgroup analysis

According to different ethnicity, genotyping method, and so on, we carried out subgroup analyses of the relationship between thrombomodulin gene polymorphisms and the risk of VTE.

#### 2.5.8 Sensitivity analysis

Through the study of large weight of elimination effect, the sensitivity analysis was performed to test the stability of the results of meta-analysis.

#### 2.5.9 Assessment of publication biases

The funnel plots will be applied to examine the publication bias if there are over 10 eligible studies.^[26,27]^

#### 2.5.10 Ethics and dissemination

The content of this article does not involve moral approval or ethical review and would be presented in print or at relevant conferences.

## 3 Discussion

Thrombomodulin gene is located on chromosome 20 P12 (20p12) and encodes 5 specific gene domains: plant hemagglutinin-like fragment, 6 epithelial growth factor-like repetitive domains, glycosyl binding region rich in serine and threonine, transmembrane region, and cytoplasmic region. The last 3 epithelial growth factor-like structures are the binding sites of thrombin and play a very important role in the anticoagulant function of Thrombomodulin.^[28]^ Stroncek et al^[29]^ used the antithrombotic effect of inner Thrombomodulin that is overexpressed by Thrombomodulin. Raife et al^[30]^ established a mouse model, knocked 2 independent human Thrombomodulin genes into apolipoprotein E-deficient mice and propagated them, and then tested the antithrombotic and anti-inflammatory effects of human thrombomodulin in vivo.

It has been previously reported that the frequency of Thrombomodulin mutation in patients with thromboembolic disease is about 5%.^[31]^ Several polymorphisms or mutations in the coding and promoter regions of the Thrombomodulin gene have been identified. The effects of these polymorphisms on the level or activity of Thrombomodulin are not clear. Some reports exhibited that this polymorphism was not associated with VTE.^[17,18,21]^ However, some studies have suggested that this polymorphism is related to VTE.^[20]^

Up to now, although many studies have focused on the relationship between thrombomodulin gene polymorphism and VTE susceptibility, the accumulated evidence has not been systematically evaluated. In this study, we will conduct a systematic review and meta-analysis in terms of many research results to obtain more reliable risk association estimates and provide guidance for the prevention and treatment of VTE. The advantages of this study include the following aspects: We will include the latest literature; for the exploration of heterogeneity, we will try to avoid post-group subgroup analysis. To improve the credibility of the results, we will conduct a sensitivity analysis of each genetic model.

In summary, this study will provide the latest evidence to support the susceptibility of thrombomodulin gene polymorphism and VTE, and provide strategies for the prevention and treatment of VTE.

## Author contributions

**Data curation:** Meihao Wei and Xiaohui Xue.

**Formal analysis:** Meihao Wei and Xiaohui Xue.

**Funding acquisition:** Yan Wu.

**Methodology:** Meihao Wei.

**Project administration:** Yan Wu, Yipeng Pan.

**Resources:** Meihao Wei, Yipeng Pan.

**Software:** Meihao Wei.

**Supervision:** Xiaohui Xue, Yipeng Pan.

**Validation:** Xiaohui Xue and Yan Wu.

**Visualization and software:** Xiaohui Xue and Yan Wu.

**Writing – original draft:** Meihao Wei, Xiaohui Xue and Yan Wu, Yipeng Pan.

**Writing – review & editing:** Yan Wu, Meihao Wei, Xiaohui Xue, Yipeng Pan.
